# Sensitivity and predictive value of dysentery in diagnosing shigellosis among under five children in Zambia

**DOI:** 10.1371/journal.pone.0279012

**Published:** 2023-02-24

**Authors:** Sam Miti, Obvious N. Chilyabanyama, Caroline C. Chisenga, Mwelwa Chibuye, Samuel Bosomprah, Chisenga Mumba, Salome Chitondo, Seter Siziya, Dani Cohen, Roma Chilengi, Michelo Simuyandi

**Affiliations:** 1 Arthur Davison Children’s Hospital, Ndola, Zambia; 2 Center for Infectious Disease Research in Zambia, Lusaka, Zambia; 3 School of Medicine, Copperbelt University, Ndola, Zambia; 4 Tropical Diseases Research Center, Ndola, Zambia; 5 Department of Biostatistics, School of Public Health, University of Ghana, Accra, Ghana; 6 School of Public Health, Tel Aviv University, Tel Aviv, Israel; Kohat University of Science and Technology, PAKISTAN

## Abstract

**Background:**

Shigella is a leading cause of bacterial diarrhea morbidity and mortality affecting mainly children under five in the developing world. In Zambia, *Shigella* has a high prevalence of 34.7% in children with diarrhea and an attributable fraction of 6.7% in Zambian children with moderate to severe diarrhea. Zambian diarrhea management guidelines and the health ministry reporting tool Health Management Information System (HMIS) heavily rely on the WHO clinical classification of dysentery to potentially identify and estimate the burden of *Shigella* in children. This reliance on clinical dysentery as a proxy to shigellosis in under five children may be resulting in gross under-estimation of shigella disease burden in Zambia.

**Methods:**

We used existing laboratory and clinical data to examine the sensitivity and predictive value of dysentery to correctly identify *Shigella* infection in under five children with PCR confirmed *Shigella* infection in Lusaka and Ndola districts, Zambia.

**Results:**

Clinical dysentery had a sensitivity of 8.5% (34/401) in identifying under five children with *Shigella* by stool PCR. Dysentery was able to correctly classify *Shigella* in 34 of 68 bloody stool samples giving a corresponding positive predictive value of 50%. Of the 1087 with non-bloody diarrhea, 720 did not have *Shigella* giving a negative predictive value of 66.2%.

**Conclusions:**

Use of clinical dysentery as a screening symptom for *Shigella* infection in children under five presenting with moderate to severe diarrhea has low sensitivity and low positive predictive value respectively. Clinical dysentery as a screening symptom for *Shigella* contributes to gross under diagnosis and reporting of *Shigella* infection among under five children in Zambia. Further research is required to better inform practice on more accurate methods or tools to use in support of routine diagnosis, particularly in low middle-income settings where laboratory diagnosis remains a challenge.

## Introduction

*Shigella* causes over 250 million cases of shigellosis annually in low and middle-income countries (LMICs) and is the fourth commonest cause of moderate to severe diarrhea (MSD) causing up to 65,000 deaths in children under five years [1–4]. *Shigella* has four species—*S sonnei*, *S flexneri*, S *boydii* and *S dysenteriae*. These species vary in their tendency to cause dysentery, which is defined as blood in stool. *S dysenteriae* type 1 has traditionally been associated with bloody stools and to a lesser extent, *S flexneri* [5]. However, there has been changing *Shigella* disease epidemiology with recent studies indicating a global decline in the incidence of *S dysenteriae* type 1-the principal cause of epidemic or pandemic dysentery [2,6]. However, other serotypes of *Shigella* continue to dominate with *S*. *flexneri* becoming more prevalent in the developing world [1,2,5,6]. In Zambia, *Shigella* was a leading bacterial causes of MSD in under-five children in 2012 with high prevalence (34.7%) and high attributable fraction (6.7%) for MSD [7]. However, there is little published data on *Shigella* speciation in Zambia.

Bloody diarrhea in under five children in LMICs is largely caused by shigellosis and amoebiasis [8,9]. However, gaps remain on the sensitivity of utilizing a clinical diagnosis of dysentery to diagnose shigellosis in children. A meta-analysis by Tickell et al. demonstrated a significant and steady decline in the sensitivity of using a clinical diagnosis of dysentery to identify shigellosis in children with diarrhea over the years (1977 to 2016) [2]. Several studies from Africa and Asia have shown non-dysenteric *Shigella* as a more common presentation of *Shigella* diarrhea [2,10]. Consequently, the continued reliance on the World Health Organization (WHO) clinical dysentery to identify shigellosis may underestimate *Shigella* disease morbidity and mortality.

The Zambian Ministry of Health’s Health Management Information System (HMIS) and disease surveillance infrastructure currently relies on stool culture or clinical categorization of non-bloody verses bloody diarrhea to inform disease intelligence efforts, *Shigella* disease burden estimates and subsequently the diarrhea management protocols for children with MSD [9]. This paper examined the clinical presentation of confirmed cases of *Shigella* using PCR molecular techniques and determined the sensitivity and predictive value of dysentery in identifying shigellosis in children under five with diarrhea using the WHO Integrated Management of Childhood Illnesses(IMCI) [11] and the Zambia Standard Treatment Guidelines [12].

## Methods

### Study design and participants

We used existing clinical and laboratory data collected from a project that was aimed at evaluating the impact of a comprehensive diarrhea prevention and control program which was targeted at reducing post-neonatal, all-cause under-five mortality in Zambia. Consent was obtained from parents or guardians of the minors included in the study. Interventions included, (i) introduction of the rotavirus vaccine to the national immunization program, (ii) improved clinical case management of diarrhea, and (iii) a comprehensive community prevention and advocacy campaign on hand washing with soap, exclusive breastfeeding up to 6 months of age, and the use of ORS and Zinc. The study is described in detail elsewhere [7,13,14]. The data was collected between July 2012 and October 2013.

Briefly, children were eligible for the study if they were aged below five years; presented to a health care facility with caregiver confirmation of child having diarrhea (defined as the passing of ≥3 abnormally loose stools in the past 24 hrs). The criteria for MSD, as per IMCI guidelines was based on the following clinical features:

*Sunken eyes, loss of normal skin turgor, IV fluid administration, blood in stool, hospitalization for diarrhea or dysentery or classified by clinician as MSD*.

We obtained data from the Zambian Ministry of Health HMIS of all children under five reported to have had diarrhea in 2018–2020. We applied the *Shigella* prevalence in children under five as determined by PCR to estimate the extent of under reporting and potential missed *Shigella* in children under five with diarrhea.

### Sample collection

At each site, the child’s care giver was asked by study staff to notify them when the child indicated a need to use the toilet or after the child had produced stool in a diaper. About 10–15 mL of bulk stool was collected from each child at enrollment in sterile specimen containers, immediately after collection, samples were transported to the laboratory at 2–8°C, then samples were stored at -80°C until testing.

#### Laboratory procedures

The extraction of nucleic acid was done using bead beating in SK38 soil grinding tubes (Cat KT03961-1-006.2, Bertin Corporation, USA), incubated in NucliSENS® easyMAG® lysis buffer (BioMérieux, France) containing bacteriophage MS2 as an internal positive control for the assay and centrifuged. After centrifugation, the supernatant was then used for nucleic acid extraction using the commercially available QIAamp MinElute Virus Spin Kit (Qiagen, Germany) with the nucleic acid eluted at -20°C until testing the next day.

We used the PCR based multiplex qualitative Luminex x-TAG® gastrointestinal pathogen panel, according to the manufacturer’s instructions for pathogen identification in stool samples collected. The kit detects 15 enteric pathogens (i.e. bacteria, viruses, and protozoa) simultaneously [3].

#### Statistics

For this effort reported here, the following statistical methods were employed: The sample size was calculated based on confidence interval for sample sensitivity and specificity [15–17]. Assuming a prevalence of 34.7% and a sensitivity of 8%, the sample size needed for a two-sided 95% sensitivity confidence interval with a width at the most extreme at 0.100, is 329. With the assumed prevalence of 34.7% and a sample specificity of 0.950, the sample size needed for a two-sided 95% specificity confidence interval with a width of at most 0.100, is 112. The whole sample size required so that both confidence intervals have a width of less than 0.100, is 329, considering the largest of the two sample sizes.

We summarized categorical data using proportions and determined association between baseline patient characteristics and *Shigella* positivity using chi square test. We computed the sensitivity, specificity, NPV and PPV and associated 95% confidence interval for blood in stool as a diagnostic measure for *Shigella* against qPCR as the gold standard. Based on the estimated prevalence NPV and PPV we estimated the number and proportion of *Shigella* cases misdiagnosed, using the national reported data and assuming the prevalence of *Shigella* remains the same over time. This assumption holds because there has not been any major intervention which might result in a reduction in the prevalence of *Shigella*. All statistical analyses were performed in Stata SE 16 (StataCorp, College Station, TX, USA).

#### Ethics statement

This study was approved by the University of Zambia Biomedical Research Ethics Committee, University of North Carolina at Chapel Hill Institutional Review Board, and the Zambian Ministry of Health under Reference No. 014-09-11. All study participants provided written informed consent.

## Results

### Study profile

**Fig 1:**
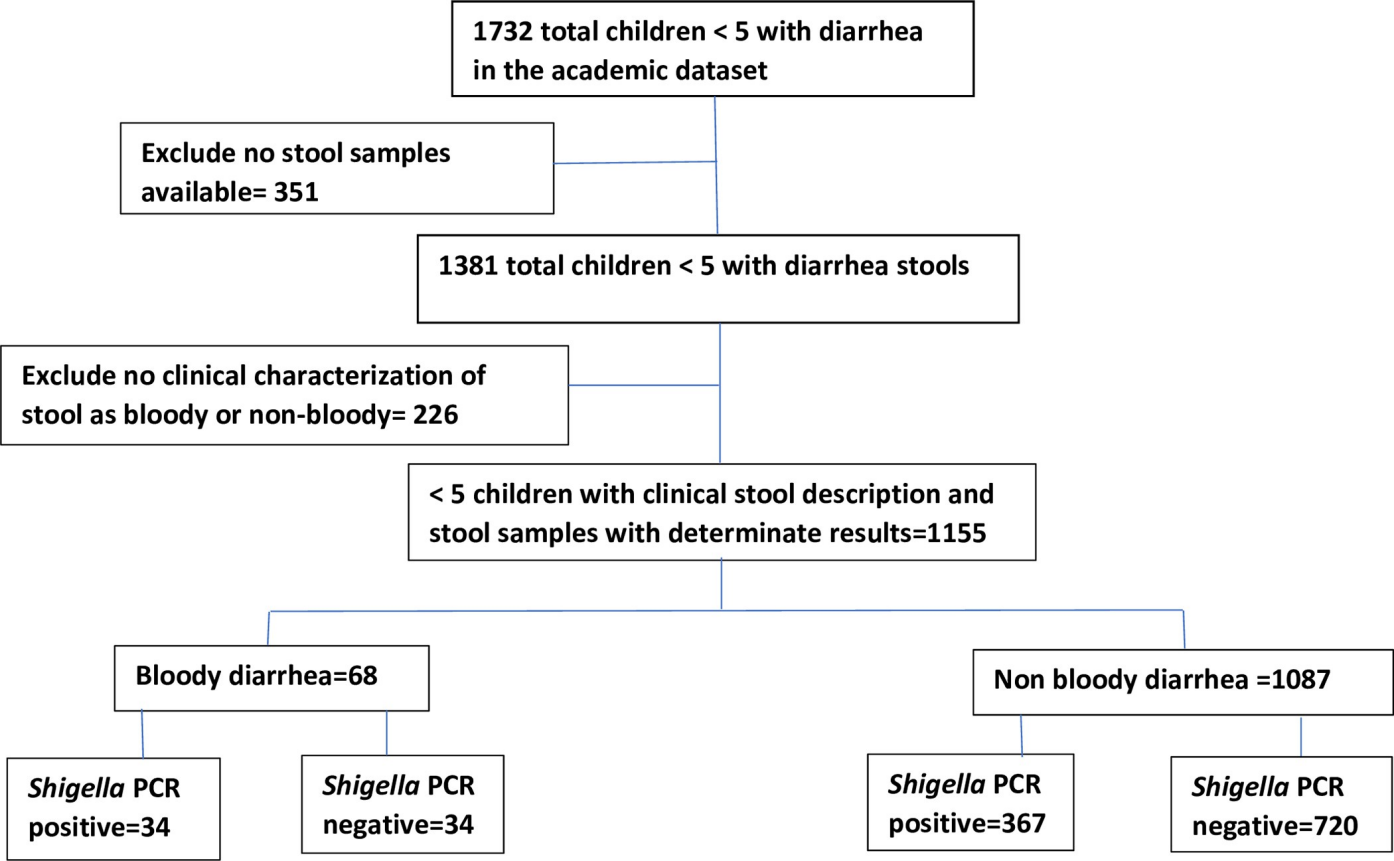
Shows the flow chart of children with *Shigella*.

1732 children under five were recorded as having diarrhea in the study database and 1381 had stool available. Only stools that met the criteria for MSD were considered. A total of 226 stool samples were excluded from the analysis because they did not have accompanying clinical characterization of “blood” or “no blood” in stool. Up to 1155 children with clinical characterization of stools by blood and PCR lab results were considered in the analysis. Fig 1 is a flow chart showing the number of children with *Shigella*.

#### Baseline characteristics

The baseline characteristics of children included age, gender and clinical symptoms at presentation to the health facility. The clinical symptoms included abdominal pain, fever and blood in stool as reported by the caregiver or observed by the health worker. Age was significantly associated with *Shigella* infection with a p-value 0.001 as shown in Table 1 below.

**Table 1:** Baseline characteristics of the children in the ACADEMIC data set.

Characteristic	n (%) of Total	*Shigella* negative, n(%)	*Shigella* positive n(%)	p value
**Age (n = 971)**				
<12 months	474(50.4)	337(71.1)	137(28.9)	0.001
12–23 months	275(29.2)	162(58.9)	113(41.1)	
24–60 months	192(20.4)	109(56.8)	83(43.2)	
**Gender (n = 1115)**				
Male	544(48.8)	366(67.3)	178(32.7)	0.116
Female	571(51.2)	358(62.7)	213(37.3)	
**Abdominal pain (n = 1115)**				
No	349(30.2)	223(63.9)	126(36.1)	0.144
Yes	686(59.4)	443(64.6)	243(35.4)	
Not reported	120(10.4)	88(73.3)	32(26.7)	
**Fever (n = 1115)**				
No	585(50.6)	386(66)	199(34)	0.407
Yes	554(48)	360(65)	194(35)	
Not reported	16(1.4)	8(50)	8(50)	
**Reported blood in stool (n = 1115)**				
No	1087(94.1)	720(66.2)	367(33.8)	0.006
Yes	68(5.9)	34(50)	34(50)	

### Prevalence, sensitivity, specificity, and predictive values results

A total of 401 were diagnosed with *Shigella* by PCR method out of the 1155 stool samples and giving a prevalence of 34.7%. Of the 401 *Shigella* positive patients, 34 had reported a history of bloody stools translating into a sensitivity of 8.5%. Using the PCR technique, a total of 754 of children with both bloody and non-diarrhea did not have shigellosis. Of these, 720 had non-bloody diarrhea out of 754 with diarrhea and no *Shigella* giving a specificity of 95.5%. A clinical diagnosis of blood in stool was able to correctly classify *Shigella* in 34 of 68 stool samples giving a corresponding positive predictive value of 50%. Further, of the 1087 with non-bloody diarrhea, 720 did not have *Shigella* giving a negative predictive value of 66.2% as shown in Table 2 below.

**Table 2:** Performance of dysentery against lab confirmed *Shigella*.

	Sensitivity (95% CI)	Specificity (95% CI)	Positive predictive value (95% CI)	Negative predictive value (95% CI)
Any blood in stool	8.5(5.9–11.7)	95.5(93.8–96.9)	50(37.6–62.4)	66.2(63.3–69.1)

### Estimated under-reporting of *Shigella* disease burden by the HMIS data reporting tool

Utilizing Ministry of Health HMIS data reports for diarrhoea in under five children for the period 2018 to 2020, there were a total of 5,857,526, 5,730,470 and 5,478,925 cases of diarrhea in 2018, 2019 and 2020 respectively. The total dysentery cases in under five children reported for the periods 2018, 2019 and 2020 were 14, 675, 13,100 and 11,332 respectively. Assuming all reported cases of dysentery were caused by shigella (over-estimation), the estimated *Shigella* cases for the reporting period was less than 1%. Applying the 34.7% prevalence of *Shigella* as detected by PCR in Chisenga et. al, the possible *Shigella* cases missed by the HMIS reporting tool are estimated at above 99% from the total diarrhoea for the 2018–2020 reporting periods. Table 3 shows the estimated under reporting of *Shigella* prevalence by HMIS if the PCR testing was utilized in the 2018–2020 HMIS data reporting period.

**Table 3:** Estimated underreporting of *Shigella* by prevalence in under five children with diarrhea in Zambia.

	Reported dysentery cases by MOH HMIS data			Expected *Shigella* cases if prevalence was 34.7%		
Year	Diarrhea (non-bloody and dysentery) OPD	Dysentery cases	Dysentery (%)	*Shigella* cases	Missed *Shigella* cases	*Shigella* cases missed (%)
2020	5,478,925	11,332	0.21%	1,901,187	1,889,855	99.40%
2019	5,730,470	13,100	0.23%	1,988,473	1,975,373	99.34%
2018	5,857,526	14,675	0.25%	2,032,562	2,017,887	99.28%

## Discussion

Our study examines the utility of clinical dysentery as a clinical proxy to diagnose *Shigella* disease in children under five with diarrhea. Clinical dysentery has been traditionally used as a clinical proxy for *Shigella* disease by the WHO and the Zambian diarrhea management guidelines. Our results show three main findings.

Firstly, our results show that although the prevalence of *Shigella* in stools of under five children presenting with diarrhea in our study was 34.7%, relaying on the clinical symptom of dysentery may be leading to significant under reporting. Our study finding that only 8.48% (34/401) of all laboratory confirmed cases of *Shigella* by stool PCR presented with dysentery, is consistent with recent literature on *Shigella* disease epidemiology [2,18,19]. This finding shows that 91.52% of under five children in Zambia who have *Shigella* infection may not present with dysentery. Recent studies including the GEMS Study have shown a changing *Shigella* disease epidemiology with a global decline in the incidence of *S dysenteriae* type 1 (traditionally the principal cause of epidemic or pandemic dysentery) and *S*. *flexneri* emerging as the predominant *Shigella* species in the developing world [1,2,5,6]. Unfortunately, there is currently little data on the seroprevalence of different species of *Shigella* in Zambia [9]. If what has been documented in other parts of the developing world is true for Zambia, the change in species prevalence and disease presentation might result in fewer children with *Shigella* infection presenting with blood in stool leading to under-reporting and ultimately suboptimal treatment of shigellosis in under-five children presenting with diarrhea.

Secondly, the sensitivity of clinical dysentery in diagnosing *Shigella* infection was very low at 8.5% while having a very high specificity of 95.5%. This important finding of low sensitivity of 8.5% speaks to the use of dysentery to estimate burden of *Shigella* disease in many countries. The presence of dysentery has been the traditional teaching about the main presentation of shigellosis [20]. The WHO diarrhea management protocols recommend the use of antibiotics in diarrhea when there is presence of blood in stool with the assumption that *Shigella* infection is the leading cause of dysentery. This continued heavy reliance on the WHO diarrhea management clinical guidelines mainly emphasizing the clinical characteristic of bloody diarrhea to identify shigellosis may be contributing to the reported under treatment of shigellosis without dysentery and the likely increased mortality in the shigellosis group without dysentery [2,10,20].

The Zambian Ministry of Health (MOH) uses the HMIS classification of diarrhea as non-bloody and bloody diarrhea [21]. Only 11,332 (0.21%) out of the total 5,478,925 diarrhea cases under five were classified as bloody diarrhea (dysentery) in 2020. It is likely that *Shigella* disease estimates in Zambia as reported by the HMIS reporting system may grossly underestimate the true burden of *Shigella* disease among children. According to Chisenga et al 2018, the prevalence of *Shigella* in under five children with diarrhea in Lusaka province was found to be 34.7% and attributable fraction for causing MSD to be 6.7% using stool PCR [7]. Applying these proportion to the MOH HMIS data in which there were 5,467,593 OPD diarrhea non bloody and 11,332 of bloody diarrhea cases in under five children in 2020 in Zambia (total diarrhea = 5,478,925) [21], the estimated number of *Shigella* cases if PCR techniques were used would have probably been about 1,901,187. However, only a paltry 11,332 dysentery cases were reported with no specific breakdown of shigellosis. Assuming all 11,332 dysentery cases are caused by *Shigella* in 2020 (gross over estimation as there are other causes such as amoebic dysentery), we can therefore extrapolate that up to 1,889,855(99%) of *Shigella* cases may not be reported in the HMIS data tool which places heavy reliance on dysentery as a symptom/proxy for *Shigella* infection (see Table 3). This agrees with our findings of low sensitivity and low predictive value of dysentery to identify *Shigella* infection in Zambian children under five who have diarrhea. This resonates well with findings from a Kenyan study in which the use of clinical symptoms to identify shigellosis was found to have low sensitivity [10]. With WHO and Zambia diarrhea management guidelines recommending antibiotic use when there is dysentery or cholera, it is likely that the majority of *Shigella* cases may be under treated based on these guidelines and may be contributing to the high mortality arising from diarrhea [22]. This study has therefore shown that over reliance on the clinical parameter of dysentery as a clinical proxy for *Shigella* infection in Zambian under five children may be leading to gross under reporting and underestimating the true burden of *Shigella* disease burden.

Thirdly, this study shows that use of clinical dysentery as a diagnostic screening symptom for *Shigella* infection has low positive and negative predictive values of 50% and 66.2% respectively. Our study results show that half of all the bloody diarrhea did not have *Shigella*. These low predictive values mean that clinicians and health care workers may not be able to place much reliance on the symptom of blood in stool to both identify or exclude under five children with *Shigella* infection. Unfortunately, Zambia like most developing countries continues to use the WHO’s clinical dysentery as a proxy to estimate the burden of shigellosis [9,10]. The clinical importance of this low positive predictive value for identifying *Shigella* disease is that without appropriate laboratory confirmation, many children with *Shigella* infection may not receive appropriate treatment.

Although stool culture is the gold standard for diagnosis of shigellosis, it is not widely available in LMICs and has poor yield compared to PCR [3]. Limitations to laboratory diagnosis of *Shigella* include limited laboratory infrastructure and consumables, inadequate microbiology culture skills/competencies, poor clinical and laboratory staff attitudes, and sample courier logistical challenges [20,22–24]. There is therefore urgent need for *Shigella* diagnostics that have low cost, high sensitivity and high specificity which can be widely available like the malaria rapid diagnostic tests [25–27]. The current reality however is that these are not yet widely available and mainly under development.

The current pragmatic approach to rational antibiotic use in diarrhea case management in which the WHO recommends antibiotic use for dysentery and cholera needs to be discussed in the light of emerging evidence [2,18,19]. Where there are diagnostic challenges and the screening symptoms which have low sensitivity and low predictive values, there is need for an appropriate balance between maintaining antibiotic stewardship and addressing mortality and morbidity due to untreated shigellosis in children. Further, there is need for point of care screening tests, diarrhea scoring systems or models with better predictive values for screening against *Shigella* infections in children under five. In the absence of in an effective *Shigella* vaccine or improved diagnostics, health care workers in LMIC will need to have a high index of suspicion for shigellosis in children with non-bloody diarrhea. The findings of this paper may refine the empiric management of diarrhea management protocols for Zambia.

### Study limitation

Although the strength of this study is its use of stool PCR data to challenge the WHO clinical dysentery symptoms as a diagnostic proxy for shigellosis in children under five children (and subsequent antibiotic use) where there is no laboratory culture, the major limitation of this study is that it was done on retrospective data limited by inconsistent collection of associated clinical parameters/symptoms. Factor analysis of the nature of diarrhea, associated fever, abdominal pain, and vomiting would have been very informative in predicting a model that can utilize clinical symptoms to predict shigellosis in under five children with diarrhea.

## Conclusion

The use of clinical dysentery as a screening symptom for *Shigella* infection in children under five presenting with moderate to severe diarrhea has low sensitivity and low positive predictive value. This contributes to under diagnosing and reporting of *Shigella* infection among under five children in Zambia. More accurate burden of disease data will be required to strengthen the case for accelerated vaccine development which holds the possibility of effective prevention of shigellosis.

### Recommendation

1. Well-designed studies be done that can evaluate symptoms associated with *Shigella* diarrhea to come up with shigella diarrhea predictive models or scoring systems for use in clinical settings.
2. Stool handling and microbiology laboratory processes be improved to increase diagnosis of shigellosis in LMICs. Additionally, point of care Rapid Diagnostic Testing (RDT) for *Shigella* are urgently needed to improve diarrhea management in LMICs.
